# A Specific Haplotype of the *MMP2* Gene Promoter May Increase the Risk of Developing Cerebral Palsy

**DOI:** 10.3390/diagnostics15243178

**Published:** 2025-12-12

**Authors:** Ana Djuranovic Uklein, Natasa Cerovac, Dijana Perovic, Nela Maksimovic, Biljana Jekic, Milka Grk, Marija Dusanovic Pjevic, Milica Rasic, Natasa Stojanovski, Milica Pesic, Ivana Novakovic, Tatjana Damnjanovic

**Affiliations:** 1 Institute of Human Genetics, Faculty of Medicine, University of Belgrade, 11000 Belgrade, Serbia; dijana.perovic@med.bg.ac.rs (D.P.); nela.maksimovic@med.bg.ac.rs (N.M.); biljana.jekic@med.bg.ac.rs (B.J.); milka.grk@med.bg.ac.rs (M.G.); marija.dusanovic-pjevic@med.bg.ac.rs (M.D.P.); milica.rasic@med.bg.ac.rs (M.R.); natasa.stojanovski@med.bg.ac.rs (N.S.); milica.pesic@med.bg.ac.rs (M.P.); ivana.novakovic@med.bg.ac.rs (I.N.); tatjana.damnjanovic@med.bg.ac.rs (T.D.); 2 Clinic for Neurology and Psychiatry for Children and Youth, 11000 Belgrade, Serbia; 3 Faculty of Medicine, University of Belgrade, 11000 Belgrade, Serbia

**Keywords:** *MMP2*, promoter polymorphisms, hypoxic–ischemic encephalopathy, cerebral palsy, MRI

## Abstract

**Background/Objectives:** Hypoxic–ischemic encephalopathy (HIE) is a common neurological outcome of perinatal asphyxia, with cerebral palsy (CP) being the most severe lasting effect. Perinatal brain injury activates the immune system and induces the release of inflammatory mediators. Matrix Metalloproteinases (MMPs) play a crucial role in neuroinflammation and neurodegeneration. This study explored the potential link between *MMP2* promoter polymorphisms and the development of CP in children with a history of perinatal asphyxia. **Methods:** We enrolled 212 patients (130 males and 82 females) with documented perinatal asphyxia, who underwent a comprehensive neurological assessment and neuroimaging, including ultrasound and magnetic resonance imaging (MRI). We genotyped the *MMP2* promoter polymorphisms rs243866, rs243865, and rs243864 using real-time polymerase chain reaction. Haplotype frequencies were calculated using Haploview software. **Results:** As expected, patients with HIE are more likely to develop CP (*p* = 0.000). In a study of 104 patients who developed CP, the frequencies of the A (rs243866), T (rs243865), and G alleles (rs243864) were nearly twice as high compared to those without CP (*p* = 0.008, *p* = 0.019, and *p* = 0.008, respectively). Haplotype analysis supported these findings, showing that the ATG haplotype was significantly more common among patients who developed CP (*p* = 0.004). Additionally, in patients with MRI-confirmed brain damage, the ATG haplotype was more frequently observed (*p* = 0.019). **Conclusions:** The ATG haplotype of the *MMP2* promoter may indicate a risk factor for developing cerebral palsy (CP) in patients who experience perinatal asphyxia and could serve as a potential diagnostic predictor of CP.

## 1. Introduction

Perinatal asphyxia happens when there is insufficient blood flow or gas exchange to or from fetal tissues during the critical periods just before, during, and after birth [1]. In developed countries, it affects about 2 per 1000 births, while in developing countries, the rate can be up to 10 times higher due to limited access to healthcare. About 15–20% of affected infants may not survive the neonatal period, and around 25% of survivors are likely to face severe, lasting neurological deficits. Hypoxic–ischemic encephalopathy (HIE) is a common neurological outcome of perinatal asphyxia, with cerebral palsy (CP) being the most serious consequence [1,2,3].

The pathogenesis of HIE and CP is complex and not fully understood. After a hypoxic–ischemic insult, brain injury occurs in two phases: the acute phase, characterized by necrotic processes due to ischemia that lead to irreversible damage, and the latent (reperfusion) phase, during which apoptotic processes extend into previously unaffected. Susceptibility to ischemia varies with gestational age, leading to distinct injury patterns. Term infants usually experience gray matter damage, leading to laminar cortical necrosis, while preterm infants mostly suffer white matter damage, causing periventricular leukomalacia [4]. These injuries are key factors in CP, the most common cause of lasting motor disability in children [5].

Historically, CP was diagnosed based on clinical observation of delayed motor development and altered muscle tone. However, advances in imaging techniques, such as cranial ultrasound and magnetic resonance imaging (MRI), have led the American Academy of Neurology and the Child Neurology Society to recommend confirming clinical diagnoses with imaging [6]. MRI is now considered the gold standard for assessing hypoxic–ischemic injury, and combining genetic and imaging data may enhance understanding of individual vulnerability and improve early diagnosis [7,8].

Matrix metalloproteinases (MMPs) are zinc-dependent enzymes that play a vital role in both normal brain function and hypoxic–ischemic injury [9]. In the central nervous system, MMPs are involved in synaptic signaling, maintaining the blood–brain barrier, neuroinflammation, and neuronal loss. Besides HIE, the expression of these enzymes increases in neuroinflammatory and neurodegenerative diseases such as Parkinson’s, Alzheimer’s, and multiple sclerosis, with elevated levels potentially worsening brain injury [10].

Research has focused on MMP-2 and MMP-9, which are crucial for maintaining blood–brain barrier integrity and for processes such as angiogenesis and remyelination. Elevated MMP-2 levels are associated with white matter damage, while MMP inhibition may help reduce neuronal loss and support recovery [11,12].

The *MMP2* gene encodes the proenzyme MMP-2, and its expression may be affected by promoter polymorphisms such as −1575G/A, −1306C/T, and −790T/G. These *MMP2* single nucleotide polymorphisms (SNPs) are associated with ischemic stroke, intracerebral hemorrhage, cardiovascular diseases, and certain cancers [13,14]. However, there is limited research on the *MMP2* gene polymorphisms as risk factors for developing CP. Therefore, this study aims to investigate the potential association between *MMP2* promoter SNPs (−1575G/A (rs243866), −1306C/T (rs243865), and −790T/G (rs243864)) and CP onset, comorbidities, and neurodiagnostics in children with a history of perinatal asphyxia.

## 2. Materials and Methods

### 2.1. Participants

This retrospective prospective study was conducted from 2017 to 2024 at the Clinic of Neurology and Psychiatry for Children and Youth in Belgrade, Serbia, as well as the Institute of Human Genetics, Faculty of Medicine, University of Belgrade. During this period, a total of 212 children were diagnosed with perinatal asphyxia according to standard guidelines [1]. All participants underwent comprehensive neurological evaluations and were classified using the Sarnat and Sarnat system [15]. Neuroimaging assessments, including ultrasound and MRI, were used to confirm HIE and IVH/periventricular leukomalacia. For patients included retrospectively, data on neuromotor assessment and additional diagnostics were obtained from existing medical records, while prospectively included patients were monitored from the neonatal period at regular 3–6 month intervals for at least 2 years. The diagnosis of CP was made based on standard criteria [5]. All patients ranged in age from neonates to 16 years old.

Additionally, epidemiological data were collected, including information on gender, gestational age (GA), birth weight, and Apgar scores at five minutes (AS 5′). The main inclusion criterion was the presence of perinatal asphyxia. Exclusion criteria were other established causes of neonatal encephalopathy, such as preeclampsia, intrauterine growth restriction, congenital anomalies, maternal or fetal infection, uncontrolled diabetes or thyroid disease of the mother, severe neonatal jaundice (kernicterus), maternal or fetal toxic exposure, and maternal thrombophilia confirmed by genetic testing. Also, children with established genetic causes of cerebral palsy, such as monogenic disease or chromosomal aberrations, were excluded. Furthermore, children who developed other specific neurodevelopmental disorders, aside from cerebral palsy, were not included in the study. The research received approval from the Ethical Committee of the Clinic of Neurology and Psychiatry for Children and Youth (No. 1-109/2) and the Ethical Committee of the Faculty of Medicine at the University of Belgrade (No. 1322/IV-5).

### 2.2. DNA Extraction and Genotyping

The molecular genetics analysis was conducted at the Institute of Human Genetics, Faculty of Medicine, University of Belgrade. DNA was isolated from peripheral blood leukocytes using the salting-out method [15]. We genotyped three SNPs in the *MMP2* promoter region: rs243866 (−1575 G/A), rs243865 (−1306 C/T), and rs243864 (−790 T/G) by real-time polymerase chain reaction (RT-PCR). TaqMan^®^ SNP genotyping assays were performed according to the manufacturer’s instructions, utilizing the ABI 7500 real-time PCR machine from Applied Biosystems (Foster City, CA, USA).

### 2.3. Statistical Analysis

Statistical analyses are performed using SPSS version 16.0 (SPSS Inc., Chicago, IL, USA). The statistical differences in genotype and allele frequencies among the groups were compared using the chi-square test. Continuous clinical data were analyzed using either Student’s *t*-test or the Kruskal–Wallis test, depending on the distribution of the variables. The association between *MMP2* promoter polymorphisms and the analyzed variables (HIE, CP, ultrasound, and MRI) was explored using the odds ratio calculator (VassarStats). The correlation was analyzed using the Spearman rank correlation test. Haplotype analysis was performed using the “Confidence Intervals for LD” method in Haploview software 4.2.

## 3. Results

### 3.1. Clinical Characteristics of the Study Group

Our study included 212 children, 130 males, and 82 females. The average gestational age (GA) at birth was 33.9 weeks (±4.9), with a median of 33 weeks, and a range from 24 to 42 weeks. The average birth weight was 2358.4 g (±1023.3), with a median of 2100 g, ranging from 640 g to 5740 g. The average Apgar score at the fifth minute (AS 5′) was 6.38 (±2.2), with a median score of 7 on a scale of 0 to 10.

Intraventricular hemorrhage was observed in 33 children (15.6%), while HIE was present in 102 children (48.1%). Cerebral palsy was diagnosed in 104 children (49.1%), and 108 children (50.9%) did not have CP.

Table 1 summarizes the clinical characteristics, risk factors for CP, comorbidities, and neuroimaging of CP. There were no significant differences in gender, sepsis, or neonatal convulsions prevalence between children with and without CP. Similarly, GA, birth weight, and AS 5′ showed no significant differences. However, children with CP had higher rates of IVH (*p* < 0.001, OR = 6.0, 95% CI = 2.36–15.27) and HIE (*p* < 0.001, OR = 15.6, 95% CI = 7.93–30.73) compared to those without. All children with CP exhibited developmental delays (*p* < 4.39 × 10^−18^). The prevalence of epilepsy was also significantly higher in these children (*p* < 0.001, OR = 14.9, 95% CI = 6.00–37.19). Pathological findings on ultrasound (*p* < 0.001, OR = 21.4, 95% CI = 10.38–44.04), and MRI (*p* < 0.001, OR = 18.1, 95% CI = 8.62–38.14) were outstanding predictors of CP development.

### 3.2. Genotype and Allele Frequencies

Table 2 shows the frequencies of *MMP2* promoter polymorphisms in our study group. The frequencies of all analyzed polymorphisms were in Hardy–Weinberg equilibrium.

### 3.3. Associations of MMP2 Genotypes with Neurological Outcomes

Table 3 shows the distribution of *MMP2* promoter genotypes and allele frequencies in relation to HIE and CP diagnoses. In children with HIE, the frequencies of the −1575 G/A (GA+AA), −1306 C/T (CT+TT), and −790 T/G (TG+GG) genotypes were nearly twice as high as in children without HIE (*p* = 0.010, OR = 2.1, 95% CI = 1.19–3.33; *p* = 0.033, OR = 1.8, 95% CI = 1.05–3.24; *p* = 0.018, OR = 2.0, 95% CI = 1.13–3.51, respectively). However, the frequencies of the −1575 A, −1306 T, and −790 G alleles did not differ significantly between children with or without HIE.

Children diagnosed with CP exhibited significantly higher frequencies of the −1575 G/A (GA+AA), −1306 C/T (CT+TT), and −790 T/G (TG+GG) genotypes compared to children without CP (*p* = 0.001, OR = 2.7, 95% CI = 1.54–4.90; *p* = 0.002, OR = 2.4, 95% CI = 1.36–4.21; *p* = 0.001, OR = 2.5, 95% CI = 1.44–4.49, respectively). Additionally, the −1575 A, −1306 T, and −790 G alleles were more common in children with CP than in those without (*p* = 0.008, OR = 2.0, 95% CI = 1.21–3.20; *p* = 0.019, OR = 1.8, 95% CI = 1.12–2.98; *p* = 0.008, OR = 1.9, 95% CI = 1.20–3.10). Our findings suggest that the genotypes and alleles of the *MMP2* promoter SNPs are strongly linked to the development of CP.

The distribution of CP subtypes was as follows: 48.5% had spastic quadriparesis, 21.4% spastic hemiparesis, 9.7% spastic diplegia, 12.6% hypotonic, 1.9% had dyskinetic (extrapyramidal), and 5.8% had mixed CP. The frequencies of risk genotypes for the analyzed promoter polymorphisms within each CP subtype were as follows: 44.0% to 46.0% in spastic quadriparesis, 36.4% to 40.9% in spastic hemiparesis, 70.0% in spastic diplegia, 69.2% in hypotonic CP, 50.0% in dyskinetic (extrapyramidal) CP, and 66.7% in mixed CP. There were no significant differences in genotype frequencies based on the CP subtype.

*MMP2* promoter polymorphisms demonstrated a similar pattern of association with neuroradiological findings as they did with neurological outcomes (Table 4). A marginal statistical significance was observed for the −790 T/G polymorphism, indicating that TG+GG genotypes were more often found in patients with detectable ultrasound pathology. Children with MRI-verified central nervous system damage had a significantly higher prevalence of the −1575 G/A (GA + AA), −1306 C/T (CT + TT), and −790 T/G (TG + GG) genotypes compared to children with normal MRI results (*p* = 0.002, OR = 2.6, 95% CI = 1.42–4.89; *p* = 0.003, OR = 2.5, 95% CI = 1.36–4.61; *p* = 0.001, OR = 2.7, 95% CI = 1.48–5.02, respectively). Additionally, the −1575 A, −1306 T, and −790 G alleles were more prevalent in children with confirmed CNS damage (*p* = 0.038, OR = 1.8, 95% CI = 1.06–2.98; *p* = 0.037, OR = 1.7, 95% CI = 1.03–2.81; *p* = 0.007, OR = 2.0, 95% CI = 1.19–3.27, respectively). Overall, the genotypes and alleles of the *MMP2* promoter exhibited a strong correlation with MRI findings.

### 3.4. Haplotype Analysis

**Figure 1:**
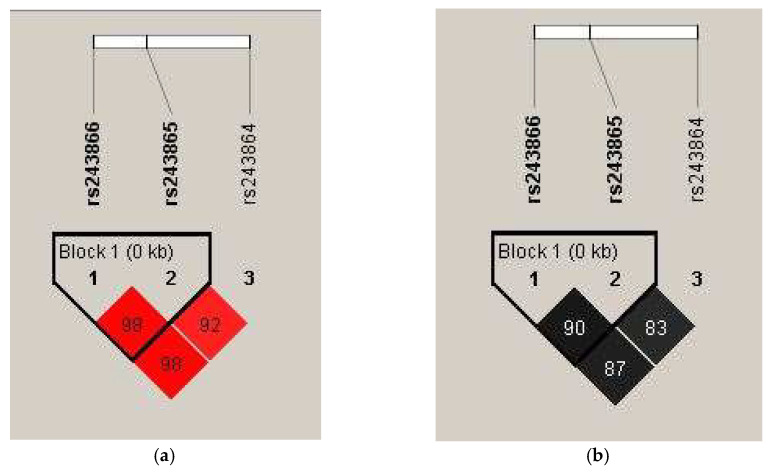
Values of (**a**) D′ and (**b**) r^2^ for the analyzed *MMP2* gene polymorphisms. In panel (**a**), the intensity of the red color and the numbers in the fields represent the D′ values—a bright red indicates D′ = 100% with a logarithm of the odds (LOD) greater than 2. A decrease in color intensity reflects a lower D′ value. In panel (**b**), different shades of gray and their corresponding numbers illustrate r^2^ values, with darker shades indicating higher r^2^ values.

Polymorphisms −1575 G/A, −1306 C/T, and −790 T/G, within the *MMP2* gene, exhibit linkage disequilibrium (LD). Therefore, haplotype analysis for these SNPs was also conducted (Table 5). Figure 1 presents the obtained D′ and r^2^ values demonstrating this relationship. A strong haplotype block was identified between the −1575 G/A and −1306C/T polymorphisms of the *MMP2* gene using the “Confidence Intervals for LD” method [16].

Haplotype analysis was performed on 212 patients. A significant difference was found in the frequencies of the ATG and GCT haplotypes of the *MMP2* gene polymorphisms when comparing patients with cerebral palsy to those without. These frequencies also correlated with MRI findings (Table 4). Patients without CP were more likely to have the GCT haplotype, while those with CP were more likely to have the ATG haplotype (*p* = 0.022 and *p* = 0.004, respectively). Additionally, the GCT haplotype was more common in patients with normal MRI results, whereas the ATG haplotype was more prevalent in patients with pathological MRI findings (*p* = 0.013 and *p* = 0.019, respectively). The ATG haplotype is identified as a genetic risk factor for developing CP and shows a strong correlation with MRI-detected pathology. As expected, a significant link exists between these pathological MRI findings and the development of CP (r = 0.617, *p* < 0.001, 95% CI = 0.519–0.699), and haplotype analysis suggests that the ATG haplotype could serve as a diagnostic predictor of CP.

## 4. Discussion

Cerebral palsy is often described as an umbrella diagnosis because it covers a wide range of conditions with different causes. Genetic factors contributing to CP can be viewed from multiple angles and may vary greatly. Current research shows that significant genetic alterations, like monogenic diseases or chromosomal abnormalities, are more common causes than previously thought. Clinical features of cerebral palsy are linked to copy number variants in 4% or more individuals, while single-nucleotide variants or indels are present in at least 14% [17]. However, in most cases, the genetic aspect remains unrecognized or absent, as environmental factors—especially perinatal asphyxia—are considered enough to explain the condition. Interestingly, some children who suffer severe perinatal brain injuries such as HIE or IVH develop lasting effects like CP, while others do not. Similarly, some children with mild perinatal asphyxia still go on to develop CP later. Most likely, these cases are due to a multifactorial mechanism, where certain genetic variants, combined with hypoxic brain injury, influence the risk of lasting neuronal damage and changes in brain structure. Conversely, some genetic variants might act protectively. Recognizing these variants in patients with CP is vital for prevention and for providing optimal clinical care, including targeted treatments.

In this study, we examined clinical characteristics, neuroimaging findings, and *MMP2* gene polymorphisms in relation to CP development after perinatal asphyxia.

Prematurity and low birth weight are recognized risk factors for CP. Our results showed no significant differences in gestational age or birth weight between groups with and without CP. Interestingly, children without CP had a slightly lower average gestational age than those with CP. Although prematurity has long been considered a major risk factor for brain injury, recent evidence suggests that the immature brain may be somewhat more resistant to hypoxia–ischemia than the term brain. Gunn et al. demonstrated that the preterm brain exhibits greater neuroplasticity and tolerance to oxygen deprivation [18]. Since all our patients experienced perinatal asphyxia, Apgar scores did not differ significantly between groups, though AS 5′ values were slightly higher among children without CP, indicating less severe hypoxic insult.

As expected, HIE emerged as one of the most significant contributors to CP development. HIE was notably more common in the CP group, reinforcing its well-known role as a primary cause of motor disabilities [19,20]. One study reported that in term neonates, HIE is the main mechanism that damages deep gray nuclei and corticospinal tracts, leading to a dyskinetic form of CP later in life [21]. Volpe noted that in preterm infants, immature autoregulation, systemic hypoxia, and reperfusion-related inflammation contribute to the selective vulnerability of the periventricular white matter, increasing the risk of CP [22]. Our findings support this model: children with HIE were significantly more likely to develop CP, confirming that the severity of early hypoxic–ischemic injury is critical for long-term outcomes.

Intraventricular hemorrhage was also more frequent among children with CP in our study. This finding aligns with previous research indicating that IVH, particularly when followed by periventricular leukomalacia or gliosis, strongly predicts CP development [23]. Although the number of IVH cases in our cohort was limited, the trend confirms that hemorrhagic and ischemic lesions often coexist in the pathogenesis of CP.

Developmental delay and epilepsy were notably more frequent in the CP group. Although neonatal convulsions appeared more frequently in the CP group, the difference was not statistically significant in our cohort. Neonatal convulsions often precede epilepsy diagnosis, suggesting that seizure activity reflects the severity of brain damage rather than being an isolated manifestation. Glass et al. reported that neonatal seizures and later epilepsy correlated with extensive cortical injury on MRI [24].

Cranial ultrasound and MRI revealed abnormalities in a significant proportion of children with CP, demonstrating a strong correlation between neuroimaging findings and CP development. These results agree with those of Horber et al. and Novak et al., who recognized MRI as the gold standard for detecting brain lesions and predicting outcomes [5,25]. Cranial ultrasound remains an essential first-line imaging modality in neonates due to its accessibility and ability to identify common hemorrhagic and ischemic lesions, but further evaluation with MRI is often required to fully characterize parenchymal injury, especially when subtle hypoxic–ischemic changes are suspected [26]. A limitation of our study is that not all participants underwent MRI, including some with abnormal ultrasound results, which limited the ability to confirm lesions fully.

Metalloproteases are vital for maintaining the extracellular matrix, a process that involves carefully regulated, dynamic protein synthesis and degradation. This process occurs slowly under stable conditions but escalates during inflammation, tissue injury, or heightened mechanical stress [9]. The functional effects of *MMP2* promoter polymorphisms are not fully understood. Price et al. found that the C to T transition at −1306 disrupts Sp1 transcription factor binding (CCACC box), significantly lowering promoter activity. Additionally, the A allele of the −1575 SNP is expected to reduce the binding affinity of transcription factors like NF-κB and AP-2 compared to the G allele [27]. This is supported by in silico models such as SNP Function Prediction [28], HaploReg [29], and JASPAR [30], suggesting that these SNPs could affect transcription factor binding and chromatin states. While the minor allele frequencies of these SNPs may decrease promoter activity and potentially offer protective effects in cases of *MMP2* overexpression, the overall impact of these changes remains complex and is still being studied.

Our results suggest that *MMP2* promoter polymorphisms may affect neurological outcomes after perinatal asphyxia. Specifically, the −1575 A, −1306 T, and −790 G minor alleles were all linked to CP development. This finding aligns well with MRI pathological results, as the most reliable neuroimaging diagnostics for confirming the permanent effects of hypoxic brain injury underlying CP. Furthermore, haplotype analysis showed that the ATG haplotype, which includes these three alleles, is significantly more common in children with CP and abnormal MRI findings. In contrast, the GCT haplotype appears to have a protective effect. Our study is the first of its kind to look at the relationship between the development of CP and these genetic variations in infants who experienced hypoxic injury during the prenatal period. O’Callaghan et al. performed a case–control study with multivariable analysis of candidate SNPs selected for their association with inflammation or other previously reported risk factors for cerebral palsy in both the mother and the child. Among other analyzed SNPs, *MMP2* rs243865 showed association with CP, although statistical significance was lost after correction for multiple testing. The distribution of genotype frequencies resembles our findings for this SNP, with the CC genotype being more frequent in the control group [31]. To the best of our knowledge, this is the only study exploring the promoter *MMP2* SNPs in association with CP:

However, MMP expression, including *MMP2*, has been investigated in patients with CP at both the protein and mRNA levels. Thus, in several studies, the concentration of different MMPs in serum or amniotic fluid was measured, providing significant indirect evidence. One study demonstrated that higher concentrations of MMP-8 in amniotic fluid were significantly associated with an increased risk of CP by age 3, supporting the hypothesis that increased extracellular matrix remodeling during pregnancy may predispose to perinatal brain injury [32]. Salah et al. reported higher levels of serum MMP-9 in hypoxic–ischemic newborns, which correlated with the severity of neonatal encephalopathy [33]. Furthermore, Maqsood et al. reported significantly elevated serum MMP-9 levels and moderately elevated MMP-2 concentrations in children with CP, suggesting an active inflammatory process and blood–brain barrier dysfunction [34]. Recently, Nemska et al. demonstrated increased expression of several metalloprotease genes, particularly *MMP2*, in tendon tissue from individuals with different forms of CP. Interestingly, MMP-2, also known as gelatinase A or type IV collagenase, is the most abundantly expressed MMP in tendons [35]. Despite being caused by a non-progressive injury to the developing brain, CP leads to ongoing changes in the musculoskeletal system. These changes may manifest as spasticity, dyskinesia, dystonia, or hypertonia, often leading to fixed contractures—permanent shortening and stiffness of the muscle-tendon unit that reduces joint mobility [36].

Studies in adults have reported similar molecular pathways. The same *MMP2* promoter polymorphisms have been associated with increased susceptibility to Alzheimer’s disease, vascular dementia, and Parkinson’s disease, likely due to enhanced transcriptional activity and blood–brain barrier permeability [12,37]. These parallels suggest that MMP-driven neurovascular remodeling may represent a shared mechanism across age groups—from neonatal HIE to late-life neurodegeneration [38].

However, reports regarding the role of MMP-2 in hypoxic–ischemic injury are often contradictory. Some studies in both animal and human subjects suggest that, after injury, MMP-2 levels remain stable, whereas MMP-9 is more consistently elevated [39]. Other studies indicate that both MMP-2 and MMP-9 increase, but their peak levels and the duration of elevated values differ. Administration of an anti-inflammatory agent led to inhibition of MMP-9 during the acute phase of ischemic damage and activation of MMP-2 during the delayed phase of injury, during recovery [40]. Further studies are needed to clarify the exact neuroprotective and neurodegenerative mechanisms of MMP-2 because its proteolytic activity is most likely time-dependent.

Our study faces limitations due to a small sample size. To enhance the generalizability of our findings, they should be validated in an independent cohort. Furthermore, it is essential to consider other environmental and confounding factors. Perinatal asphyxia is a key environmental factor in the development of CP. While the study’s exclusion criteria reduce the impact of other known perinatal injuries, some confounding factors remain, such as varying postnatal conditions and the effects of early treatment and developmental interventions. Additionally, as the SNPs are in the gene’s regulatory region, numerous epigenetic modifiers, which remain to be investigated, may also influence the results.

## 5. Conclusions

The integration of clinical, imaging, biochemical, and genetic data allows a more complete understanding of CP pathogenesis. Environmental triggers, such as hypoxia, hemorrhage, and infection, act synergistically with *MMP2* promoter variants to enhance matrix degradation and vascular permeability. Early identification of such variants, in combination with MRI and biochemical markers, could improve risk prediction and enable timely neuroprotective interventions.

Our data show that specific SNPs in the *MMP2* promoter—namely the −1575 A, −1306 T, and −790 G alleles, either individually or as part of the ATG haplotype—are associated with the development of CP and positive MRI findings in children who experienced an acute hypoxic–ischemic event during birth. Therefore, these polymorphisms could potentially serve as diagnostic predictors of serious neurological outcomes of perinatal asphyxia.

## Figures and Tables

**Table 1:** Clinical characteristics of the participants.

Variable		With CP *N* (%)/X ± SD	Without CP *N* (%)/X ± SD	*p* Value
Clinical trait	Gender M/F	68/36	62/46	0.233
	GA (weeks)	34.1 ± 4.8	33.9 ± 4.9	0.847
	Birth weight (g)	2346.5 ± 1002.4	2369.9 ± 1047.9	0.869
	AS 5′	6.2 ± 2.2	6.6 ± 2.2	0.223
Risk factors for CP and comorbidities	HIE	81 (79.4)	21 (19.8)	**0.000**
	IVH	27 (26.5)	6 (5.7)	**0.000**
	Developmental delay	104 (100)	59 (54.6)	**0.000**
	Sepsis	8 (8.1)	10 (9.8)	0.685
	Neonatal convulsions	37 (36.6)	30 (28.0)	0.185
	Epilepsy	48 (47.5)	6 (5.7)	**0.000**
Pathological findings in neuroimaging	Ultrasound	84 (79.2)	15 (15.2)	**0.000**
	MRI	68 (81.9)	20 (20.0)	**0.000**

Abbreviations: CP—cerebral palsy; M—male, F—female; GA—gestational age; AS 5′—Apgar score at 5th minute; HIE—hypoxic–ischemic encephalopathy; IVH—intraventricular hemorrhage; MRI—magnetic resonance imaging.

**Table 2:** Frequencies of genotypes and alleles for *MMP2* promoter polymorphisms in our study group.

SNP (rsID)	Genotypes/Alleles	Frequency *n* (%)
−1575 G/A (rs243866)	GG	133 (62.7)
	GA	73 (34.5)
	AA	6 (2.8)
	G	339 (80.0)
	A	85 (20.0)
−1306 C/T (rs243865)	CC	130 (61.3)
	CT	74 (34.9)
	TT	8 (3.8)
	C	334 (78.8)
	T	90 (21.2)
−790 T/G (rs243864)	TT	128 (60.4)
	TG	76 (35.8)
	GG	8 (3.8)
	T	332 (78.3)
	G	92 (21.7)

Abbreviations: SNP—single-nucleotide polymorphism; Note: Alleles are presented based on the genomic DNA strand. HGVS nomenclature is used for variants in the promoter region.

**Table 3:** Frequencies of genotypes and alleles for *MMP2* polymorphisms in relation to neurological outcomes.

SNP (rsID)	Genotypes/ Alleles	HIE *n* (%)		P	CP *n* (%)		*p*
		Yes	No		Yes	No	
−1575 G/A (rs243866)	GG	54 (53.5)	75 (70.8)		53 (51.0)	80 (74.1)	
	GA+AA	47 (46.5)	31 (29.2)	**0.010**	51 (49.0)	28 (25.9)	**0.001**
	G	154 (76.2)	176 (83.0)		155 (74.5)	184 (85.2)	
	A	48 (23.8)	36 (17.0)	0.111	53 (25.5)	32 (14.8)	**0.008**
−1306 C/T (rs243865)	CC	54 (53.5)	72 (67.9)		53 (51.0)	77 (71.3)	
	CT+TT	47 (46.5)	34 (32.1)	**0.033**	51 (49.0)	31 (28.7)	**0.002**
	C	153 (75.7)	172 (81.1)		150 (73.5)	180 (83.3)	
	T	49 (24.3)	40 (18.9)	0.726	54 (26.5)	36 (16.7)	**0.019**
−790 T/G (rs243864)	TT	52 (51.5)	72 (67.9)		51 (49.0)	76 (71.0)	
	TG+GG	49 (48.5)	34 (32.1)	**0.016**	53 (51.0)	31 (29.0)	**0.001**
	T	150 (74.3)	173 (81.6)		151 (72.6)	179 (83.6)	
	G	52 (25.7)	39 (18.4)	0.092	57 (27.4)	35 (16.4)	**0.008**

Abbreviations: SNP—single-nucleotide polymorphism; HIE—hypoxic–ischemic encephalopathy; CP—Cerebral palsy.

**Table 4:** Frequencies of genotypes and alleles for *MMP2* polymorphisms in relation to neuroradiological findings.

SNP (rsID)	Genotypes/Alleles	Ultrasound *n* (%)		*p*	MRI *n* (%)		*p*
		Path	Norm		Path	Norm	
−1575 G/A (rs243866)	GG	59 (55.7)	68 (68.7)		42 (50.6)	73 (73.0)	
	GA+AA	47 (44.3)	31 (31.3)	0.055	41 (49.4)	27 (27.0)	**0.002**
	G	164 (77.4)	162 (81.8)		124 (74.7)	168 (84.0)	
	A	48 (22.6)	36 (18.2)	0.319	42 (25.3)	32 (16.0)	**0.038**
−1306 C/T (rs243865)	CC	58 (54.7)	66 (66.7)		41 (49.4)	71 (71.0)	
	CT+TT	48 (45.3)	33 (33.3)	0.080	42 (50.6)	29 (29.0)	**0.003**
	C	162 (76.4)	159 (80.3)		122 (73.5)	165 (82.5)	
	T	50 (23.6)	39 (19.7)	0.402	44 (26.5)	35 (17.5)	**0.037**
−790 T/G (rs243864)	TT	56 (52.8)	65 (66.3)		39 (47.0)	70 (70.7)	
	TG+GG	50 (47.2)	33 (33.7)	0.050	44 (53.0)	29 (29.3)	**0.001**
	T	159 (75.0)	158 (80.6)		119 (71.7)	165 (83.3)	
	G	53 (25.0)	38 (19.4)	0.214	47 (28.3)	33 (16.7)	**0.007**

Abbreviations: SNP—single-nucleotide polymorphism; MRI—magnetic resonance imaging; Path—pathological; Norm—normal.

**Table 5:** Neurological outcomes, neuroradiological findings, and *MMP2* haplotype.

Haplotype	All	CP (%)		*p*	All	MRI (%)		*p*
		Yes	No			Path	Norm	
GCT	76.9	72.1	81.5	**0.022**	76.5	70.5	81.5	**0.013**
ATG	19.8	25.5	14.3	**0.004**	19.9	25.3	15.5	**0.019**
GCG	1.7	1.9	1.9	0.679	1.7	3.0	0.0	0.063
GTT	1.2	0.0	1.4	0.191	1.4	1.2	1.5	0.808

Abbreviations: CP—Cerebral palsy; MRI—magnetic resonance imaging; Path—pathological; Norm—normal.

## Data Availability

The original contributions presented in this study are included in the article. Further inquiries can be directed to the corresponding author.
